# Effectiveness of motivational interviewing and physical activity on prescription on leisure exercise time in subjects suffering from mild to moderate hypertension

**DOI:** 10.1186/1756-0500-4-352

**Published:** 2011-09-12

**Authors:** Mats Sjöling, Kristina Lundberg, Erling Englund, Anton Westman, Miek C Jong

**Affiliations:** 1 Department of Health Sciences, Mid Sweden University, Sundsvall, Sweden; 2 Husum Family Health Centre, County Council of Västernorrland, Örnsköldsvik, Sweden; 3 Research and Development Centre, County Council of Västernorrland, Sundsvall, Sweden; 4 Department of Surgical and Perioperative Sciences, Section of Anesthesiology and Intensive Care, Umeå University, Umeå, Sweden; 5 Department of Physiology and Pharmacology, Karolinska Institutet, Stockholm, Sweden; 6 Department of Nutrition and Healthcare, Louis Bolk Institute, Driebergen, The Netherlands

**Keywords:** blood pressure, health related quality of life, leisure exercise time, physical activity, prescriptive counseling

## Abstract

**Background:**

Physical inactivity is considered to be the strongest individual risk factor for poor health in Sweden. It has been shown that increased physical activity can reduce hypertension and the risk of developing cardiovascular diseases. The objective of the present pilot study was to investigate whether a combination of Motivational Interviewing (MI) and Physical Activity on Prescription (PAP) would increase leisure exercise time and subsequently improve health-related variables.

**Methods:**

This pilot study was of a repeated measures design, with a 15 months intervention in 31 patients with mild to moderate hypertension. Primary outcome parameter was leisure exercise time and secondary outcome parameters were changes in blood pressure, Body Mass Index (BMI), waist circumference, lipid status, glycosylated haemoglobin (HbA1c) and maximal oxygen uptake (VO_2 max_). Assessments of the outcome parameters were made at baseline and after 3, 9 and 15 months.

**Results:**

Leisure exercise time improved significantly from < 60 min/week at baseline to a mean activity level of 300 (± 165) minutes/week at 15 months follow up. Furthermore, statistically significant improvements (p < 0.05) were observed in systolic (-14,5 ± 8.3 mmHg) and diastolic blood pressure (-5,1 ± 5.8 mmHg), heart rate (-4.9 ± 8.7 beats/min, weight (-1.2 ± 3.4 kg) BMI -0.6 ± 1.2 kg/m^2^), waist circumference (-3.5 ± 4.1 cm) as well as in VO_2 max _(2.94 ± 3.8 ml/kg and 0.23, ± 0.34 lit/min) upon intervention as compared to baseline.

**Conclusions:**

A 15 month intervention period with MI, in combination with PAP, significantly increased leisure exercise time and improved health-related variables in hypertensive patients. This outcome warrants further research to investigate the efficacy of MI and PAP in the treatment of mild to moderate hypertension.

## Background

The Swedish National Institute of Public health considers physical inactivity to be the strongest individual risk factor for poor health in Sweden. Approximately 25-30% of middle-aged men and 10-15% of middle-aged women and teenagers (both men and women) in Sweden can be classified as being physically inactive [1]. It has been shown that increased physical activity can reduce hypertension [2] and the risk of developing cardiovascular diseases [3], diabetes, obesity, osteoporosis and depression [4]. The Swedish governing agencies thus encourage health care workers (for example; physicians, nurses, physiotherapists) to use Physical Activity on Prescription (PAP) as an instrument in disease prevention and treatment of patients [1]. It is noticed that low- to moderate intensive activity at a level of only 40% of maximal oxygen uptake VO_2 max _, is sufficient to achieve both an acute and long-term reduction in blood pressure [2]. Activities of moderate intensity correspond to physical activity at a level which leads to shortness of breath, while still being able to converse [2,5]. Therefore, unfit patients with hypertension may profit considerably from simple exercises such as walking to achieve and maintain a blood pressure reduction [2,6-8]. An important prerequisite for maintaining health benefits is that patients keep exercising [1,3]. Previous studies have shown that PAP alone is not effective in the long term, but that PAP in combination with health counseling may be more effective [9-11], for example with Motivational Interviewing (MI). MI is a specific patient-or client centered technique addressing health counseling, that was developed to increase patient's motivation for changing behavior by exploring and solving ambivalence [12]. The purpose of MI is to "negotiate" with the patient/client, to show respect for different ideas of how a change can be achieved and to avoid persuasion. This technique also contains strategies to help people developing their own inherent capability for change, for example towards increased physical activity [12].

The objective of the present pilot study was to investigate whether a combination of Motivational Interviewing (MI) and Physical Activity on Prescription (PAP) would increase leisure exercise time and subsequently improve health-related variables.

## Methods

### Study design

The pilot study was of a repeated measures design, with a 15 months intervention period and measurements at baseline and after 3, 9 and 15 months [13]. Subjects were recruited at a health centre in northern Sweden from September 2006 throughout December 2007. Approval to perform the study was obtained from the ethical review board at Umeå University (Dnr: 06-M33-1205) as well as from the head of staff at the participating Health Centre. Family doctors at the Health Centre referred patients to the study nurse when they met the inclusion criteria. Inclusion criteria were adult men and women suffering from mild to moderate hypertension (systolic blood pressure of 140-179 mmHg, diastolic blood pressure of 90-109 mmHg [14,15]). Their physical activity should correspond to less than 60 minutes per week, or performed less than two occasions per week. Subjects younger than 18 years of age, and those with dementia, diabetes or unable to understand and write the Swedish language were excluded from participation in the study.

### Outcome measurements

The primary outcome parameter of the study was leisure exercise time; Baseline values for leisure exercise time were collected on a narrative basis from the subjects during the first MI. During the entire study period of 15 months, leisure exercise time was documented in an exercise diary, including type of activity, context, intensity and duration. Secondary outcome parameters were physiological measurements such as systolic and diastolic blood pressure, Body Mass Index (BMI), waist circumference, lipid status, glycosylated haemoglobin (HbA1c) and maximal oxygen uptake (VO_2 max_). Values were assessed at baseline, and at 3, 9 and 15 months follow up. Systolic and diastolic blood pressures were measured on the right arm after 5-10 minutes of rest, with the subject either lying down or sitting in a chair (subject were assessed in the same body position at each visit). Measurements were made manually with a standardized method taking account of the subjects arm circumference in order to use the right cuff size. The study nurse performed the measurement at all visits where blood pressure was measured twice at each visit and the documented value corresponds to the mean value of the two measurements. Height was measured to the nearest 0.5 cm (with shoes removed) using a stadiometer. Weight was measured to the nearest 0.1 kg (with shoes and bulky clothing removed) using an electronic scale (a single unit; Seca Delta, Model 707; Seca, Hamburg, Germany) calibrated prior to each visit. Body mass index (BMI; kg/m^2^) was calculated. The study nurse documented Waist- and hip circumference (cm) and calculated a Waist/Hip circumference quota.

Blood analysis included; lipid status (Serum Cholesterol, Plasma-HDL, Plasma-LDL, Plasma-LDL/HDL, Plasma-Triglycerides), Plasma Glucose and glycosylated haemoglobin (HbA1c). Blood samples were obtained by vein puncture after a 12 h overnight fast. The plasma and serum samples were separated from whole blood by centrifugation [16] and were immediately analyzed with a spectrophotometric method, using an automated analyzer system (Cobas c501, Roche Diagnostics, GmbH, Mannheim, Germany). HbA1c was measured by a fluid chromatography method - HPLC (Mono S 5/50 GL, GE Healthcare Europe, Freiburg, Germany).

In addition to leisure exercise time and physiological parameters, Subjective Health Related Quality of Life (HRQoL) was assessed with the Short Form 36 questionnaire (SF36) [17,18] at baseline and at 15 months follow up. Furthermore, maximal oxygen uptake (VO_2 max _, l/min, ml/kg) was estimated using the Åstrand test [19] at baseline and at 15 months follow up as a measure of aerobic fitness.

### Intervention

Subjects accepting participation in the study were invited to a series of four MI's with the study nurse. In the study, the motivational interviewing technique as developed by Miller and Rollnick [12] was used. In addition subjects also received PAP [1,9,20]. The study nurse involved was trained in the MI technique and the concept of PAP. At the first MI, data on previous disease, current medication, use of tobacco and food habits and leisure exercise time were documented. Subsequent MI's were performed after 3, 9 and 15 months. The MI's were aimed to develop the individual subject's notions and ideas regarding physical activity and exercise, thereby supporting them to set reachable goals within their horizon. PAP's was prescribed individually, based on the subjects' present level of physical activity, as stated by the subjects, and the notion and believe in their own capacity. A mutual goal for each subject in the study was to reach a moderate intensity level of physical activity for at least 30 minutes a day. Moderate intensity corresponds to physical activity at a level which leads to shortness of breath, while still being able to conversate. Subjects were advised to pursue physical activity on an individual level or to participate in group activities of at least for 60 minutes once a week. The subjects also received written information on physical activities arranged by different associations in their neighborhood area. All subjects were provided with an exercise diary were they noted their daily physical activity regarding type, intensity and duration of activity as well as occurring circumstances to explain changes of activity pattern.

During the MI's at three, nine and 15 months follow-up, completed exercise diaries were examined by the project nurse conjointly with the subject. In the MI's the nurse asked open ended questions concerning how the prescribed physical activity had proceeded and if there had been set backs, obstacles or success. A reflective synthesis was made by the nurse from the exercise diaries and the interview in order to motivate and promote the subject to continue with his/her prescribed activities if they were poorly motivated to pursue the previous prescription, a new individualized PAP was issued.

### Statistical methods

A one-way repeated measure test of variance (R-ANOVA) was conducted in order to explore the differences in the outcome measure. For the purpose of data reduction, mean values of weekly leisure exercise time were computed before being entered in the R-ANOVA. Mean week values were used in the "within group" analysis of leisure exercise time to calculate a contrast, were values from month one through 15 were compared with the baseline value. For other outcome measures; month 3, 9 and 15 were compared with the baseline value. VO_2 max _and SF-36 were analyzed using a dependent sample t-test. A test with a p-value less than 0.05 was considered statistically significant.

Analyzing data collected in repeated measures, the pair-wise mean differences were only computed when the full R-ANOVA model had a p-value of less than 0.05. In the results mean values are presented together with standard deviation, SD. Statistical analysis was made using STATISTICA version 8 [21].

## Results

In total, 64 subjects were invited to participate in the study of which 30 subjects denied participation. Of the 34 subjects entering the study, three subjects dropped out during the course of the study due to serious medical condition (n = 2) or moving out of the area (n = 1). The full set analysis included 31 subjects, 11 men and 20 women with a mean age of 61.6 years (range 43-71). Other baseline demographic characteristics with respect to marital status, smoking and use anti-hypertensive medication use are shown in Table 1.

**Table 1:** Demographic characteristics at baseline of the 31 subjects participating in the study

Gender	
Male (%)	64
Female (%)	36
Age (Mean ± SD)	61.6 ± 7.0 (range 43-71)
Marital status	
Married/Living together (%)	84
Single (%)	16
Tobacco use (smoking)	
Current user (%)	13
Previous user (%)	42
Medication for hypertension (%)	58

**Figure 1:**
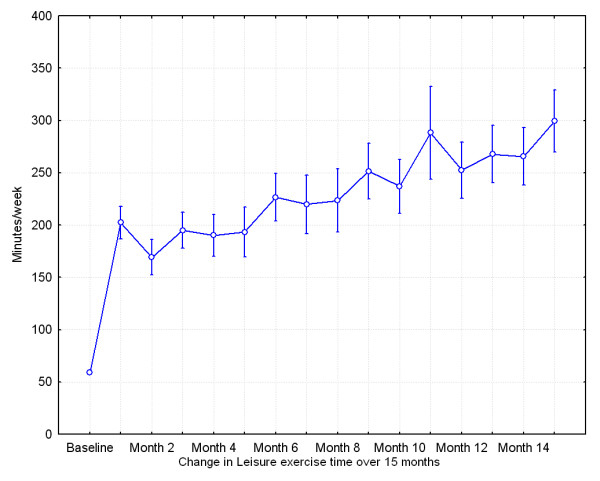
**Mean leisure exercise time per week during the study period from baseline to 15 months follow up**. Vertical bars denote +/- standard errors.

**Figure 2:**
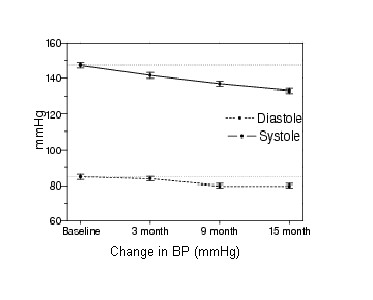
**Systolic and Diastolic blood pressure at baseline, 3 month, 9 month and 15 month follow-up**.

An overview of the main outcome parameters is given in Table 2. As noted there were significant improvements in most outcome parameters during the 15 month intervention period. At baseline all subjects had a leisure exercise time < 60 minutes per week. After 15 months intervention, leisure exercise time had significantly improved to a mean activity level of 300 minutes/week (± 165 minutes). Mean activity level increased directly in the short term at 1 month follow up, but also continue to increase during the 15 month intervention period (Figure 1). Furthermore, a total of 30 subjects (97%) had a leisure exercise time exceeding 60 minutes per week and 27 subjects (87%) had a leisure exercise time exceeding 30 minutes a day. After 15 months of intervention, reductions (R-ANOVA, p < 0.05) were observed in systolic (mean difference of -14.5 mmHg, ± 8.3) and diastolic blood pressure (mean difference of -5.1 mmHg, ± 5.8), heart rate (mean difference -4.9 beats/min, ± 8.7), weight (mean difference -1.2 kg, ± 3.4), BMI (mean difference-0.6 kg/m^2^, ± 1.2) and waist circumference (mean difference -3.5 cm, ± 4.1). With respect to blood lipid analysis, a mean reduction in serum cholesterol, 0.4 mmol/l (± 1.0) was observed, Table 2 and Figure 2.

**Table 2:** Data regarding primary outcome measures, (Mean ± SD), collected at baseline and at subsequent visits at 3, 9 and 15 months

	Baseline	3 months	9 months	15 months	**Mean difference **Baseline-15 months	p-value
Leisure exercise time (Minutes physical activity/week	< 60	195 ± 96***	251 ± 149***	300 ± 165**		< 0.001***
Blood pressure(mm Hg)						
Systolic blood pressure	147.4 ± 7.8	141.7 ± 9.8***	136.6 ± 8.3***	132.9 ± 6.7**	-14.5 ± 8.3	< 0.001***
Diastolic blood pressure	84.8 ± 8.4	83.8 ± 7.6	79.7 ± 6.6***	79.7 ± 6.4**	-5.1 ± 5.8)	< 0.001***
Heart rate (b/min)	67.3 ± 10.7	64.1 ± 10.5*	63.2 ± 6.8*	62.4 ± 6.2*	-4.9 ± 8.7	0.003**
Weight (kg)	89.7 ± 14.1	89.3 ± 14.0	89.0 ± 14.2	88.4 ± 13.6*	-1.2 ± 3.4	0.048*
BMI (kg/m^2^)	32.0 ± 4.8	31.8 ± 4.9	31.7 ± 5.0	31.4 ± 4.8*	-0.6 ± 1.2	0.001**
Waist circumference (cm)	103.2 ± 11.3	100.8 ± 11.0*	100.4 ± 10.0*	99.7 ± 10.3*	-3.5 ± 4.1	< 0.001***
Lipid status (mmol/l)						
Cholesterol	6.1 ± 1.0	6.1 ± 1.3	5.8 ± 0.9*	5.7 ± 0.9*	-0.4 ± 1.0	0.02*
High density lipoprotein cholesterol	1.5 ± 0.3	1.5 ± 0.3	1.4 ± 0.3	1.4 ± 0.4		0.6
Low density lipoprotein cholesterol	3.7 ± 0.9	3.7 ± 1.1	3.4 ± 0.9	3.4 ± 0.9		0.6
Triglycerides	1.9 ± 0.8	1.9 ± 0.9	1.8 ± 0.9	1.8 ± 0.8		0.7
						
Plasma glucose	5.4 ± 0.8	5.6 ± 1.2	5.6 ± 1.0	5.6 ± 1.1		0.35
HbA1c (%)	4.7 ± 0.6	4.7 ± 0.7	4.7 ± 0.6	4.8 ± 0.6		0.18
Maximal oxygen uptake (VO_2 max_)						Dependent sample t-test
lit/min	2.0 ± 0.4	Not measured	Not measured	2.2 ± 0.5	0.23 ± 0.34	0.001**
ml/kg	22.6 ± 4.4	Not measured	Not measured	25.6 ± 5.6	2.94 ± 3.8	< 0.001***

*p < 0.05, **p < 0.01, ***p < 0.001.

The * at the follow up values refers to the post hoc test (contrasts) where follow up at 3, 9 and 15 months was compared with baseline. The p-value and * in the last column refers to the significance test of the R-ANOVA model or from dependent samples t-test when mentioned.

Improvements were observed (dependent samples t-test, p < 0.05) in aerobic fitness-maximal oxygen uptake (VO_2 max_; ml/kg; mean difference 2.94, ± 3.8, lit/min; mean difference 0.23, ± 0.34) after 15 months compared to baseline values (Table 2). No changes were observed regarding, tobacco or alcohol habits during the 15 month intervention, although two subjects stopped using medication for hypertension.

With respect to quality of life (SF36 scores), improvements (dependent samples t-test, p < 0.05) were observed in "role physical", "bodily pain" and "vitality" after 15 months of intervention as compared to baseline (Table 3).

**Table 3:** Data regarding Health Related Quality of Life - SF 36, (Means ± SD) collected at baseline and at the subsequent visit at 15 months

	Baseline	15 months	p-value
SF-36 HRQoL			
Physical functioning	78.1 ± 19.2	81.8 ± 18.3	0.17
Role physical	64.5 ± 43.2	82.3 ± 43.2	0.04*
Bodily pain	61.0 ± 25.5	69.0 ± 27.2	0.02*
General health	65.3 ± 20.0	68.4 ± 19.8	0.15
Vitality	54.4 ± 23.5	63.4 ± 23.9	0.02*
Social functioning	82.3 ± 22.1	83.9 ± 21.0	0.63
Role emotional	68.8 ± 44.7	82.8 ± 34.3	0.61
Mental health	77.5 ± 16.5	78.7 ± 19.0	0.85

*p < 0.05.

The measures were tested with a dependent sample t-test. An increase in the different values indicates an improvement.

## Discussion

In the present pilot study we provided a lifestyle intervention, consisting of Motivational Interviewing and Physical Activity on Prescription, to subjects with mild to moderate hypertension. The intervention was successful in encouraging patients to increase leisure exercise time, both from a short and a long-term perspective. It was at least sufficient to obtain significant improvements in a large variety of health related variables, i.e. decreased systolic and diastolic blood pressure, heart rate, weight BMI, waist circumference and total cholesterol and improved maximal oxygen uptake (VO_2 max_). In line with these results, the improvement in VO_2 max _indicated that the physical fitness of the subjects was increased. The VO_2 max _values of subjects at the endpoint of the present study were comparable to normative data reported in a Canadian study [22]. The extent of leisure exercise time in the present study is in line with the recommended dose for people with hypertension, corresponding to brisk walks, Nordic walking, biking, swimming etc for at least 30 minutes, 5-7 times per week [1,5]. A recent literature review reported that, unfortunately, many studies do not reach the recommended amount of leisure exercise time (> 150 min or more per week) necessary to achieve positive health effects [23].

The observed reduction in BMI and waist circumference upon the intervention could be of clinical importance as high BMI and waist circumference, in combination with hypertension, are strong indicators of the metabolic syndrome, a cluster of symptoms that is associated with further development into cardiovascular disease and diabetes [24,25].

The observed improvement in the present study with respect to the quality of life domains "role physical", vitality and bodily pain were also observed in a study by Elley *et.al *[9]. These effects may be connected to the fact that the subjects undergoing this life-style intervention have a better aerobic fitness and by this experience specifically a higher HRQoL in those domains. However, due to the limited sample size in the Elley et.al [9] study and the present study, it is not possible to make any definite conclusions with respect to this observation.

Our results support the findings from several other studies where physical activity on prescription along with health information have a positive effect on health variables and quality of life, [26-28] In contrast to the present study as well as to the study by Elley *et.al *[9], Eriksson *et.al *[26] were unable to establish an effect on leisure exercise time. In their study the life-style intervention focused on group exercise training, diet counseling and follow-up meetings. In our present study, as well as in Elley *et.al *[9] and Kallings *et.al *[28], the intervention was primarily focused on MI and PAP as motivators for life-style change, suggesting those to be important factors for the documented improvements in leisure exercise time and related health variables. Crucial aspects in MI is that the conversation/interview relies on mutual understanding and trust between the health professional and the patient, which only has an opportunity to emerge when given sufficient time for the patient. Another aspect is that health professionals/district nurses need to increase their knowledge through education and training in MI techniques [1].

The present study was initiated as a pilot study with a small sample size without comparison to a control group. Therefore, it is not possible to draw any firm conclusions about the specific efficacy of PAP in combination with MI in hypertensive patients or to generalize the results to other groups of patients. Other (context) factors such as daily monitoring and documenting activities, as well as increased attention by the study nurse may have contributed to the beneficial effects as observed. Another limitation of the pilot study was that a substantial amount of subjects (about 50%) that were approached to participate in the study were not willing to do so. Thus a selection bias may have occurred, including subjects in the study that were highly motivated and more prone to change their lifestyle.

In healthcare systems of today, it has become more and more necessary to prioritize which diagnoses and interventions to include among the publicly funded treatment options. Treatment of hypertension will most likely, continue to be of high priority. In Sweden there is a strong trend both nationally [1] and regional [29], to promote PAP as one of the tools in the treatment plan. Despite its limitations, the present pilot study gave valuable insights in the pragmatic feasibility and possible effect size of a combination intervention with MI and PAP in the treatment of hypertensive patients. The actual MI's and PAP could be performed within the regular working time of the district nurse, indicating that the intervention can be implemented in every day practice in Healthcare Centres in Sweden. Furthermore, only 2 out of 31 subjects were unable to complete the study, leaving dropout to be a minor problem for this intervention. MI in combination with PAP, lead to a significant and sustained increase in leisure exercise time and subsequent improvement in

health-related variables. However, larger scale randomized controlled studies, including cost effectiveness, are necessary to confirm the efficacy of MI and PAP and its place in standard treatment of mild to moderate hypertension.

## Conclusions

MI, in combination with PAP, appeared to be a promising tool for health care professionals to promote a more physically active lifestyle in hypertensive patients. A 15 month intervention period, significantly increased leisure exercise time and improved health-related variables. The outcome of the present pilot study warrants further research to investigate the efficacy of MI and PAP in the treatment of mild to moderate hypertension.

## Competing interests

The authors declare that they have no competing interests.

## Authors' contributions

MS, KL and EE all participated in the design of the study. KL conducted all the motivational interviews and data collection. MS, KL and EE made the initial analysis of the data and drafted the manuscript. All authors (MS, KL, EE, AW and MCJ) had discussions about the analysis and reporting as well as in finalizing the manuscript. All authors read and approved the final manuscript.
